# Examining the Role of Quality of Institutionalized Healthcare on Maternal Mortality in the Dominican Republic

**DOI:** 10.3390/ijerph20146413

**Published:** 2023-07-20

**Authors:** Maria De Jesus, Nora Sullivan, William Hopman, Alex Martinez, Paul David Glenn, Saviour Msopa, Brooke Milligan, Noah Doney, William Howell, Kimberly Sellers, Monica C. Jackson

**Affiliations:** 1 School of International Service & Center on Health, Risk, and Society, American University, Washington, DC 20016, USA; 2 Data Science, Purdue University, West Lafayette, IN 47907, USA; 3 Statistics and Data Science, The University of Texas at San Antonio, San Antonio, TX 78249, USA; 4 Agricultural and Environmental Sciences, McGill University, Montreal, QC H3A 0G4, Canada; 5 Mathematics and Statistics, American University, Washington, DC 20016, USA; 6 Mathematics, Temple University, Philadelphia, PA 19122, USA; 7 Mathematics, University of Maryland College Park, College Park, MD 20742, USA; 8 Mathematics and Statistics, Georgetown University, Washington, DC 20057, USA

**Keywords:** reproductive health, Dominican Republic, maternal mortality

## Abstract

The main study objective was to determine the extent to which the quality of institutionalized healthcare, sociodemographic factors of obstetric patients, and institutional factors affect maternal mortality in the Dominican Republic. COM-Poisson distribution and the Pearson correlation coefficient were used to determine the relationship of predictor factors (i.e., hospital bed rate, vaginal birth rate, teenage mother birth rate, single mother birth rate, unemployment rate, infant mortality rate, and sex of child rate) in influencing maternal mortality rate. The factors hospital bed rate, teenage mother birth rate, and unemployment rate were not correlated with maternal mortality. Maternal mortality increased as vaginal birth rates and infant death rates increased whereas it decreased as single mother birth rates increased. Further research to explore alternate response variables, such as maternal near-misses or severe maternal morbidity is warranted. Additionally, the link found between infant death and maternal mortality presents an opportunity for collaboration among medical specialists to develop multi-faceted solutions to combat adverse maternal and infant health outcomes in the DR.

## 1. Introduction

A high maternal mortality ratio (MMR) poses a significant threat to women’s health in developing countries. The World Health Organization (WHO) definition of MMR is the “number of maternal deaths during a given time period per 100,000 live births during the same time period”. [1]. Maternal mortality refers to deaths due to complications from pregnancy or childbirth. Maternal mortality is largely preventable. In 2015, the WHO published, “Strategies toward ending preventable maternal mortality (EPMM)”, a set of approaches and goals to help countries across the globe improve the safety and well-being of mothers in childbirth. The EPMM includes achieving three main targets by 2030: to reduce the global MMR to under 70/100,000 live births, to reduce each nation’s MMR by at least two-thirds of their 2010 baseline level, and to prevent any nation from reaching an MMR greater than 140/100,000 live births [2].

From 2000 to 2017, the global maternal mortality ratio declined by 38%, representing a drop from 342 deaths to 211 deaths per 100,000 births (live births), which translates into an average annual rate of reduction of 2.9% [1]. While this figure represents a global decline in MMR, the improvement in maternal health has occurred unevenly, with the greatest burden of maternal deaths (94%) occurring in developing countries [1]. The MMR of the Dominican Republic (DR) has increased over the period between 2000 and 2017, rising from 80 in 2000 to 95 in 2017 [3,4].

The DR presents an unusual relationship between its maternal mortality rate and access to maternal care. High rates of access to antenatal visits are typically associated with lower MMR [5,6]. In 2019, 97% of patients in the DR attended at least one prenatal care visit during pregnancy by skilled health personnel, 97.7% of patients have institutionalized deliveries (i.e., in a health facility), and 98.2% of deliveries are attended by skilled health personnel [7]. Still, the DR’s MMR remains persistently greater than typical indicators would predict.

Such widespread access to prenatal care and institutionalized deliveries raises questions regarding the quality of care provided to patients at healthcare facilities. The current study seeks to explain the maternal mortality paradox in the Dominican Republic and examines the extent to which the quality of institutionalized care, as well as relevant sociodemographic and institutional factors, might explain DR’s MMR.

### 1.1. Quality of Care

Miller et al. demonstrated that adequate access to institutionalized care alone in the DR does not negate the poor quality of care received by obstetric patients at the healthcare facility [8]. In the DR, health care facilities, especially public hospitals, are overcrowded and under-resourced [8,9]. Patients often share beds with one another, even when such beds are contaminated with bodily fluids [6,7,8]. Hospitals lack the resources to provide patients with hospital gowns, leaving them entirely unclothed [10,11]. While bed-sharing presents a physical health risk, the psychological stress and emotional degradation of being unclothed presents an additional mental health risk, highlighting larger problems regarding the inhumane treatment of maternal patients in public hospitals [9]. Provider incompetence is another significant predictor of maternal health outcomes [6,7,8,9,10,11]. Providers report a lack of experience that also contributes to poor quality healthcare, noting that students and residents are often assigned to care for laboring mothers [8,10].

In the DR context, providers often neglect patients in need of urgent medical attention while overmedicalizing the births of low-risk patients, performing routine episiotomies and unnecessary Cesarean-sections (C-sections) [8,10,11]. C-sections are life-saving procedures when performed appropriately; however, when performed frequently and unnecessarily, they pose a risk to the patient’s health [12]. One recent study found that obstetric patients may elect to have C-sections on account of fearing the standard of care provided in care facilities, especially the lack of pain medication available for labor and birth [13].

### 1.2. Obstetric Violence

The poor quality of care provided to maternal patients in the DR has been referred to as “obstetric violence”. This term includes dehumanized care, disrespect and abuse, or mistreatment during childbirth, as well as different forms of physical and emotional abuse and neglect endured by maternal patients in poor hospital facilities [7,9,14,15,16,17]. Essentially, obstetric violence violates the human rights of women “to the highest attainable standard of health, which includes the right to dignified, respectful health care” and it both reflects and perpetuates social and gender inequities [18,19]. It is an urgent issue that affects women giving birth in clinical settings throughout the world and is a key driver of global inequitable maternal and child health outcomes [7,18,20].

### 1.3. Potential Sociodemographic Factors Associated with MMR

The DR has a high teenage pregnancy rate, with approximately 20.5% of adolescents aged between 15 and 19 becoming pregnant [10,21,22]. A study conducted across developing nations found that teenage patients are twice as likely to die from causes related to pregnancy and childbirth than older patients [23]. Adolescent pregnancy is a significant and preventable concern as it relates to the maternal mortality ratio.

Additionally, single motherhood is a potential sociodemographic factor affecting MMR. A UK study found that single patients struggle to meet recommended dietary and transportation needs compared to their married counterparts [24]. Single patients also suffer from higher rates of anxiety, depression, paranoia, and suicidal thoughts, all of which might contribute to maternal mortality.

Previous research suggests the employment status of patients also affects maternal health outcomes. While not studied extensively in the DR, a study conducted in Argentina demonstrated that both infant and maternal health improves with a nation’s economy. In conditions of economic destitution, factors such as malnutrition, when combined with the psychological stress of pregnancy, yielded worse maternal health outcomes [25]. It is likely that women’s employment status as well as the nation’s employment rate impact MMR in the DR.

### 1.4. Current Research

This study aims to measure a variety of factors to further examine the role between quality of care, demographic factors, and maternal health outcomes. Factors measured include (1) single mother birth rates, (2) teenage mother birth rates, (3) vaginal birth rates, (4) unemployment rates, (5) hospital bed rates (bed rate), (6) infant death rates, (7) and sex of child rates. Additionally, this study examines infant death rate because of a demonstrated link between infant death and maternal mortality in the developing world [26].

## 2. Methods

### 2.1. Variables

This study is a cross-sectional study in which data about the Dominican Republic population were used to infer the relationship between maternal mortality and demographic factors and quality of care factors. The variable “MMR” represents the number of maternal mortalities from 2015 to 2019 in a province divided by the number of births of the province [27,28]. This method of calculating the MMR is fit for our analysis of MMR. In contrast, the WHO calculated the MMR per 100,000 births and of the entire country. The “Unemployment Rate’’ refers to the number of unemployed people actively searching for labor in the province in 2019 divided by the economically active population in the province in 2019 [29,30]. The economically active population is the population that is employed plus the population of those that actively looked for a job in 2019 in each region of the Dominican Republic [30]. The “Single Mother Birth Rate” is the total number of births to single mothers in a province from 2015 to 2019 divided by the total number of births in the province from 2015 to 2019 [28]. The “Bed Rate” refers to the number of hospital beds within a province in 2020 divided by the total population of the province in 2020 [31,32]. The “Vaginal Birth Rate” represents the number of births in the province that were vaginally delivered (not C-section) in 2012 (newer data were not available) divided by total births in 2012 [28,33]. The 2012 data may not reflect newer data. There may be a discrepancy between the most current and the 2012 data used in this study, which might affect the significance of this explanatory variable. “Infant Death Rate” represents the total number of infant mortalities in a province in 2019 divided by the total number of births in the province in 2019 [27,28]. The “Sex of Child Rate” was calculated by dividing the total number of births of male babies by female babies per province from 2015 to 2019 [28]. “Teen Birth Rate” was calculated by dividing the total number of births by mothers aged below 20 years from 2015 to 2019 by total number of births in the province from 2015 to 2019 [28]. “Birth Rate” was calculated by dividing the number of births in a province in 2015 to 2019 by population in that province from 2015 to 2019 [28].

This study took provincial data instead of nationwide data to capture the variability in demographic variables across the DR. The provincial data for maternal mortality varied too widely year by year to accurately measure the MMR. Given that the MMR between 2015 and 2019 only fluctuated by at most 2% [34], combining sets of years represented the best approach to an accurate analysis of factors influencing MMR.

### 2.2. Pearson Correlation

The Pearson correlation was used to assess correlation between all the independent variables with its corresponding *p*-values.

### 2.3. Conway-Maxwell-Poisson Regression

To predict the response (maternal mortality rate) from the explanatory variables, the Poisson regression was used. All programming was carried out in R 4.2.1. The four assumptions of the Poisson regression include: (1) the response variables are in count per unit of space or time as described by a Poisson distribution, (2) the observations must be mutually independent, (3) the mean of the Poisson random variable is equal to its variance, and (4) the log of the mean rate (log(λ)) is a linear function of x. The model used in this study fulfills all assumptions except for the one that the assumption that the mean is equal to the variance. In this study we ran the COM-Poisson as an exploratory statistic for MMR as the response variable and found that the intercept was not significant. This indicated normal dispersion and demonstrated that the Poisson regression would effectively model the data despite the mean and variance being unequal.

The COM–Poisson distribution captures the Poisson distribution as a special case that can be used when the data have over- or under-dispersion. This study used COM-Poisson to analyze infant death rate as the response variable because the dispersion intercept is significant and negative, which indicates under-dispersion. The R statistical package COMPoissonReg was used for the COM-Poisson model [35].

To measure the error, this study used an offset of total births by province. The offset adjusts the variation in total births across provinces mostly caused by population differences. The offset alters the response variable to be the log of “per total births” in each province.

In addition to analyzing MMR, this research produced an additional section electing to study infant mortalities as a response variable. Basic analytics, as well as the COM-Poisson regression method, were similarly used to observe a meaningful predictive value. Measuring infant mortality may uncover other predictors for MMR that maternal health research may further investigate.

### 2.4. Patient and Public Involvement

Patients or the public were not involved in the design, conduct, reporting, or dissemination plans of our research.

## 3. Results

The study examined the relationship of maternal mortality with potential explanatory variables and the relationship of infant death rate with the same explanatory variables. Table 1 includes the descriptive statistics for each variable.

**Figure 1:**
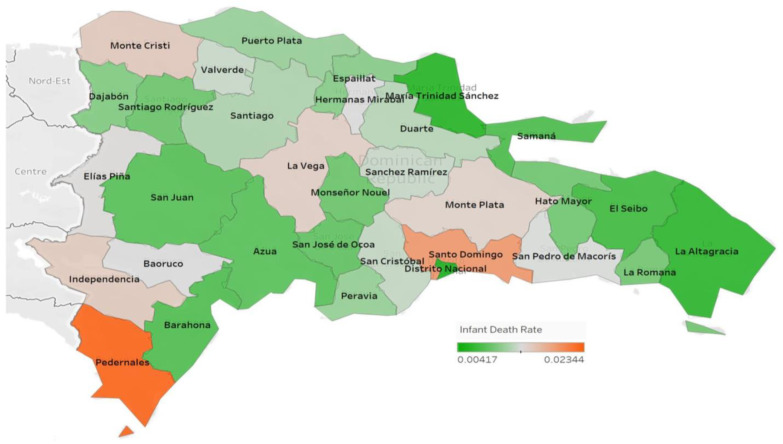
Infant death rate by province.

**Figure 2:**
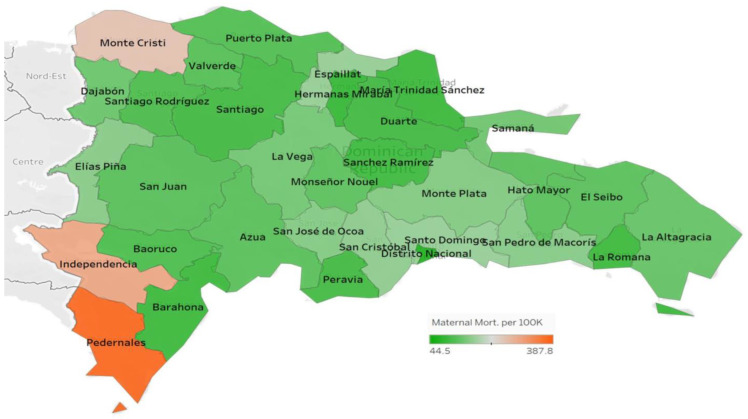
Maternal mortality rate by province.

Figure 1 and Figure 2 represent the proportions of the infant death rate and maternal mortality rate by province, respectively, in the Dominican Republic across all 31 provinces and the National District (Distrito Nacional). The infant death rate was lowest in El Seibo at 6.4 × 10^−5^ and highest in Elías Piña at 5.4 × 10^−4^. The maternal mortality rate was lowest in Hermanas Mirabal at 4.35 × 10^−5^ and highest in Independencia at 2.04 × 10^−4^.

### 3.1. Pearson Correlation

Pearson correlation coefficients were obtained for all variables considered in the analysis. Table 2 presents the coefficients for the correlations. Two variables are considered correlated if the coefficient is 0.70 or greater. According to this analysis, the single mother birth rate and teen birth rate were considered moderately correlated (r = 0.738, *p*-value < 0.001). Additionally, birth rate and single mother birth rate were considered highly correlated (r = 0.982, *p*-value < 0.001). Further, birth rate and teen birth rate were moderately correlated (r = 0.706, *p*-value < 0.001).

### 3.2. COM-Poisson Regression

The study used a Poisson regression to analyze the relationship between maternal mortality ratio and the predictor variables of teen birth rate, unemployment rate, single mother birth rate, sex of child rate, bed rate, vaginal birth rate, and infant death rate. The regression was run once with all the data. Table 3 outlines the results.

Using a significance level of 0.10, the results from the analysis suggest that at least one of the explanatory variables was a significant predictor of maternal mortality. Such variables are single mother birth rate (estimate = −9.5072, *p* = 0.008917), vaginal birth rate (estimate = 1.4974, *p* = 0.000385), and infant death rate (estimate = 24.7191, *p* = 0.069920).

While the variables teen birth rate, sex of child rate, unemployment rate, and bed rate were not significant predictors for total maternal mortality, the variables single mother birth rate, infant death rate, and vaginal birth rate were significant predictors for maternal mortality (*p* < 0.10). Specifically, the results demonstrate that as the single mother birth rate increases, the maternal mortality rate decreases with an estimate of −9.5072; as the vaginal birth rate increases, the maternal mortality rate increases with an estimate of 1.4974; and as the infant death rate increases, the maternal mortality rate increases with an estimate of 24.7191.

This study also assessed the relationship between infant death rate and the same predictor variables. The regression was run once with all the data. Table 4 outlines the results.

Using a significance level of *p* = 0.10, the results from the analysis suggest that at least one of the predictor variables was a significant predictor of infant mortality. The significant predictors of infant death rate were single mother birth rate (estimate = −15.3186, *p* < 0.001), unemployment rate (estimate = −4.57, *p* < 0.001), and vaginal birth rate (estimate = 0.9538, *p* < 0.01). Sex of child rate, bed rate, and teen birth rate were poor predictors of infant death rate (*p* > 0.10).

## 4. Discussion

This study investigated predictors of maternal mortality in the Dominican Republic.

First, the study showed that as the single mother birth rate increases, maternal mortality rate decreases, which suggests that single motherhood improves maternal health outcomes in the DR (Table 3). Based on previous research suggesting a correlation between single motherhood and lower socioeconomic status, we predicted that single mothers would experience worse health outcomes as lower socioeconomic status is typically correlated with a high maternal mortality ratio [36,37,38,39,40]. There are a few plausible explanations for this unexpected result. First, it is possible that single mothers must rely upon a greater and more expansive social support network than their married counterparts. Such social networks can provide emotional support (care and concern), informational support (advice and guidance), and instrumental support (resources, financial and otherwise), which have been demonstrated to improve health outcomes in various sectors of the population [41,42,43,44,45]. It is reasonable to suggest that these findings may translate to maternal health specifically, especially given evidence finding that Central and South American single mothers tend to form close social bonds with their communities [39,40]. Further research is needed to investigate this phenomenon as it applies to single mothers in the Dominican Republic specifically.

Additionally, the study results demonstrated that as the vaginal birth rate increases, maternal mortality increases, which suggests that C-sections (as opposed to vaginal births) improve maternal health outcomes in the DR (Table 3). This result may reflect the poor quality of care provided to patients in Dominican hospitals [6,7,8,9]. Although we would predict higher rates of C-sections (and lower rates of vaginal births) would increase maternal deaths, it is possible that a scarcity of resources causes providers to neglect patients having vaginal births and focus on those having C-sections [10,11]. While routine C-sections pose a threat to a patient’s health, it is possible that the poor hospital conditions in the DR force providers to channel limited resources into the more medically complicated procedures, thus neglecting vaginal births.

The study findings also showed a lack of correlation between maternal mortality and the bed rate, teenage birth rate, and unemployment rate variables, indicating that they bear no effect on maternal health outcomes (Table 2). This result diverges from the previous research supporting the theory that MMR would decrease as these variables increase. It is possible these widespread unexpected results are due to poor quality of national and hospital maternal outcome data and data reporting. Given that public hospitals are understaffed and under-resourced, it is not unreasonable to suggest certain factors go unreported. For example, it is possible that, in a stressful environment staffed with overworked providers, factors such as the age of mother are incorrectly recorded (if recorded at all). It is also possible that other operational approaches would better capture the ways in which these risk factors correlate with maternal health outcomes. For example, replacing the dependent variable of “maternal mortality ratio” with maternal near-miss (MNM) or severe maternal morbidity (SMM), which include the experiences of surviving patients, might better capture how these risk factors affect the trauma and physical stress endured by certain segments of the population. A study that measures maternal health outcomes and experiences, rather than maternal deaths, might yield results more consistent with the previous literature. Such research might consider psychological stress and trauma endured by patients as poor maternal health outcomes.

An unexpected result from the COM-Poisson distribution included the lack of a relationship between infant death rates and maternal death rates (Table 4). This result refutes the theory that children who lose their mother shortly after birth experience an increased risk of child mortality [26]. Lastly, the Pearson correlation coefficient confirms information gathered by previous research as it demonstrates that there is a significant portion of women giving birth in the DR who are single mothers, teenagers, or both (Table 2) [10,21,22].

Two study limitations are that the National Office of Statistics of the Dominican Republic only had bed rate data available from the year 2020 and vaginal birth rate data from 2012; while the other variables were calculated with data from the years 2015–2019.

### Implications for Interventions to Improve Maternal Mortality Incomes

One intervention strategy to improve maternal mortality outcomes is a modified obstetric early warning system (MOEWS), which uses a system of color-coding to identify “clinically-deteriorating” patients [37]. The MOEWS can improve the monitoring of patients in a low-resource setting. It has been implemented and met with success in the United States, United Kingdom, and Zimbabwe [37,38,39]. Being that low availability of resources, insufficient attention paid to patients, and understaffing at hospitals are key causes for concern in the Dominican Republic, MOEWS might provide a means of mitigating these forms of obstetric violence.

Others advocate for a data collection method that relies not only upon data pertaining to maternal deaths, but also data pertaining to near-maternal deaths, known as “maternal near-misses” (MNMs), or sometimes as instances of “severe maternal morbidity” (SMM) as mentioned previously [46,47,48]. Collecting MNM and SMM data would allow for more feedback from patients, a benefit that solely measuring maternal deaths does not allow [38,49]. Additionally, this strategy highlights issues within the healthcare system that might otherwise be overlooked, such as assigning under-qualified or emotionally-abusive personnel to obstetric patients [38]. In the DR, where measurements of obstetric violence are highly reliant upon qualitative data from interviews with patients, measuring SMM and MNM may help develop the best strategies for improving facilities.

## 5. Conclusions

The results of this study warrant further research into the ways in which poor quality of care affects maternal health outcomes in a broader sense. Additional research regarding how poor quality of care may affect data reporting and analysis is warranted. The study results also demonstrate how the discourse around the quality of maternal care must transcend the measurement of individual-level experiences, and instead extend to the ways in which support systems can buffer poor quality of care. The study also revealed a link between infant death and maternal mortality, which can be interpreted as an opportunity for collaboration between researchers from multiple medical specialties to develop multi-faceted solutions to combat poor maternal and infant health outcomes in the DR. If poor maternal care influences both children and parents, there is an imperative for medical researchers and advocates to combine resources and improve maternal care for the benefit of many.

## Figures and Tables

**Table 1:** Descriptive statistics of rates per 10,000 for quality-of-care variables, demographic variables, infant mortality, and maternal mortality, Dominican Republic, 2015–2019.

	Minimum	Maximum	Median	Mean	Std. Deviation
MMR	0.435	2.04	0.865	0.926	0.362
Infant deaths	0.638	5.38	2.96	2.98	0.935
Unemployment rate	143.4	1112.0	568.2	619.0	274.3
Teen birth rate	32.3	388.9	216.7	214.4	71.8
Single mother birth rate	419.5	1479.3	834.6	865.8	217.9
Bed rate	4.54	6.58	9.38	12.4	18.4
Sex of child rate	9021.9	10,679.4	10,395.1	10,350.5	282.4
Vaginal birth rate	2590.0	8056.0	5858.5	5957.6	1190.0

**Table 2:** Pearson correlation coefficients and *p*-values of considered variables, Dominican Republic, 2015–2019. The upper diagonal represents the correlation and the lower diagonal represents the *p*-value.

	MMR	Teen Birth Rate	Un- Employment Rate	Single Mother Birth Rate	Sex of Child	Bed Rate	Vaginal Birth Rate	Infant Death Rate	Birth Rate
MMR		−0.249	−0.206	−0.299	−0.311	0.112	0.514	0.539	−0.299
Teen birth rate	0.169		−0.022	0.738	0.067	0.340	−0.076	−0.366	0.706
Un- employment rate	0.258	0.905		−0.047	0.112	0.071	−0.333	−0.383	−0.0386
Single mother birth rate	0.096	0.000	0.800		0.251	0.164	−0.051	−0.529	0.982
Sex of child rate	0.083	0.715	0.543	0.166		−0.024	−0.238	−0.385	0.227
Bed rate 2020	0.541	0.057	0.701	0.371	0.896		−0.070	−0.047	0.196
Vaginal birth rate	0.003	0.680	0.062	0.781	0.190	0.702		0.425	−0.0789
Infant death rate	0.001	0.040	0.030	0.002	0.030	0.797	0.015		−0.522
Birth rate	0.0962	6.48 × 10^−6^	0.834	0	0.211	0.283	0.668	0.002	

**Table 3:** Maternal mortality, Poisson regression results, Dominican Republic 2015–2019.

Coefficients:				
	Estimate (Log Scale)	Std. Error	z Value	Pr (>|t|)
(Intercept)	−11.7347	2.8316	−4.144	3.41 × 10^−5^
Teen birth rate	−9.8370	8.2326	−1.195	0.232128
Unemployment rate	1.6980	1.7900	0.949	0.342821
Single mother birth rate	−9.5072	3.6353	−2.615	0.008917
Sex of child rate	3.8285	2.5806	1.484	0.137925
Bed rate	172.6341	161.6696	1.068	0.285602
Vaginal birth rate	1.4974	0.4218	3.550	0.000385
Infant death rate	24.7191	13.6387	1.812	0.069920
(Dispersion parameter for Poisson family taken to be 1)				

**Table 4:** Infant death, COM-Poisson regression results, Dominican Republic.

Coefficients:				
	Estimate	Std. Error	Z Statistics	*p* Value
(Intercept)	−5.5387	1.4233	−3.8913	9.97 × 10^−5^
Teen birth rate	2.2327	4.8370	0.4616	0.6444
Unemployment rate	−4.5737	0.8780	−5.2090	1.898 × 10^−7^
Single mother birth rate	−15.3186	1.4121	−10.8479	2.041 × 10^−27^
Sex of Child Rate	0.5055	1.3343	0.3788	0.7048
Bed rate	−0.0366	94.5633	−0.0004	0.9997
Vaginal birth rate	0.9538	0.3091	3.0857	0.002031
MMR	0.0186	880.4206	0.0000	1
Dispersion intercept	−0.0534	0.0270	−1.9795	0.04776

## Data Availability

The data sets used and/or analyzed in the current study are available from the corresponding author upon reasonable request.
